# A novel retractor-assisted closed reduction combined with percutaneous pinning fixation for the treatment of elderly distal radius fractures: a retrospective cohort study

**DOI:** 10.1186/s13018-021-02556-6

**Published:** 2021-06-26

**Authors:** Bin Zhao, Wenqian Zhao, Isaac Assan, Zhenji Li, Rongxiu Bi

**Affiliations:** 1 Postdoctoral Research Station, Shandong University of Traditional Chinese Medicine, 4655#, University Road, Changqing District, Jinan, 250355 Shandong Province China; 2 Department of Orthopedics, Shouguang Hospital of Traditional Chinese Medicine, 3353#, Shengcheng Street, Shouguang City, 262700 Shandong Province China; 3 Department of Geriatric Medicine, Qilu Hospital of Shandong University, 107#, Wenhuaxi Road, Jinan, 250012 Shandong Province China; 4 Department of Traditional Chinese Medicine, The People’s Hospital of Shouguang City, 1233#, Jiankang Street, Shouguang City, 262700 Shandong Province China; 5 School of International Education, Weifang Medical University, 7166 Baotong West Street, Weicheng District, Weifang City, 261053 Shandong Province China; 6 Department of Cardiovascular, Shouguang Hospital of Traditional Chinese Medicine, 3353#, Shengcheng Street, Shouguang City, 262700 Shandong Province China; 7 Department of Orthopedics, Affiliated Hospital of Shandong University of Traditional Chinese Medicine, 16369#, Jingshi Road, Jinan, 250014 Shandong Province China

**Keywords:** Steinmann pin retractor, Percutaneous pinning fixation, Distal radius fracture

## Abstract

**Background:**

Percutaneous pinning fixation (PCP) has been used for the treatment of distal radius fractures for decades, especially in the elderly with fragile soft tissue. However, achieving and maintaining a sound anatomic reduction before PCP is difficult if we use the manipulative reduction method alone. Our study innovatively applied the Steinmann pin retractor for closed reduction combined with PCP, to provide a new protocol for the treatment of distal radius fractures.

**Methods:**

From March 2017 to July 2018, 49 patients out of 57 that met the inclusion criteria but not the exclusion criteria were included in our retrospective cohort study. Sixteen patients were treated with Steinmann pin retractor-assisted closed reduction combined with PCP (S-PCP), and 19 patients were treated with the manipulative reduction combined with PCP (M-PCP), and 14 patients were treated with the manipulative reduction combined with cast splint (M-C). All these patients received a positive postoperative radiological and clinical evaluation.

**Results:**

All the patients were followed up for a minimum of 2 years. The radiological parameters in each group improved significantly postoperative (posttreatment). In the S-PCP group, the values of radial height (postoperative, 13.33±1.74 mm; the first follow-up, 13.27±1.81mm; last follow-up, 13.16±1.76mm) and ulnar variance (postoperative, −0.10±1.29mm; the first follow-up, −0.05±1.27mm; last follow-up, −0.12±1.09mm) significantly improved as compared to the M-PCP and M-C groups. While the patients in the M-C group experienced significant re-displacement at the first and last follow-ups, in the S-PCP group, the range of wrist motion including extension (89.94±5.21%), radial deviation (90.69±6.01%), and supination (90.25±5.87%); ulnar deviation (89.81±5.82%) and QuickDASH score (2.70±3.64); and grip strength (92.50±5.59%), pronation (90.50±6.04%), and modified Mayo wrist score (90.94±4.17, the excellent rate reached up to 75%) also improved as compared to the M-PCP group, M-C group, or both groups at the last follow-up.

**Conclusion:**

S-PCP improves fracture reduction and wrist function and can serve as an effective method for A_2_(AO/OTA) and A_3_ type of distal radius fractures in the elderly with limited dorsal comminution, including intra-articular fractures with displacement less than 2mm.

**Supplementary Information:**

The online version contains supplementary material available at 10.1186/s13018-021-02556-6.

## Introduction

Distal radius fractures are common upper limb fractures with up to 18% incidences in the elderly [1]. It usually occurs in postmenopausal women [2]. Distal radius fractures in the elderly are mostly caused by low-energy injuries from osteoporosis disease complications [3]. Most of them are unstable fractures that must be treated with a surgical procedure. These elderly patients usually come in with pre-existing hypertension, diabetes, or other complicated diseases which makes the soft tissue at the fracture end very fragile. This brings about risks to open reduction and internal fixation (ORIF). However, fractures in the elderly seldom involve the radiocarpal articular surface, which provides a perfect indication for percutaneous pinning fixation (PCP). PCP has the following advantages: low-cost treatment, minimally invasive, and early removal of fixation at 6 weeks postoperative. The challenge this protocol faces is that achieving and maintaining a sound anatomic reduction before PCP when the manipulative closed reduction method alone is used is difficult. For this reason, this new protocol (Steinmann pin retractor-assisted closed reduction) which has not been previously reported was used before PCP (S-PCP).

To evaluate the efficacy of this new protocol, we retrospectively made a comparison with manipulative reduction combined with PCP (M-PCP) or cast splint (M-C) protocol. The comparisons were made in these lights: measurement of radiological parameters, including radial height, radial inclination, ulnar variance, volar tilt, and radial shift. Clinical evaluation included a visual analog scale (VAS); the modified Mayo wrist score; and the Quick Disabilities of the Arm, Shoulder, and Hand (*Quick*DASH) score. They were evaluated over the follow-up period.

## Material and method

### Patients

From March 2017 to July 2018, 49 patients out of 57 that met the inclusion criteria but not the exclusion criteria were included in our retrospective cohort study. All the patients had distal radius fractures from the department of orthopedics of Shouguang Hospital of Traditional Chinese Medicine.

### Inclusion criteria

Patients with distal radius fracture who had received the M-C were enrolled. Based on the AAOS guideline for operative fixation [4], patients with distal radius fracture displaying a post-reduction dorsal tilt of >10°, or radial shortening of >3mm, who had received the PCP were enrolled. Patients having associated intra-articular fractures but with a displacement of less than 2mm, osteoporosis, and ulnar styloid process fractures were also included.

### Exclusion criteria

This retrospective cohort study excluded intra-articular fractures with a displacement of more than 2mm. Oblique volar fractures, die-punch fractures, or dorsal comminution involving more than one-third of the diameter of the articular surface were excluded. The following were also excluded: aged less than 50 years; patients with open fractures, bilateral fractures, and multiple fractures; lost to follow-up postoperatively.

### Demographic and group information

S-PCP group involved 16 patients, 12 female and 4 male cases respectively. Eleven were on the left and 5 on the right, 8 A_2_ types and 8 A_3_ types. Eight were complicated ulnar styloid fractures. The average age was 64.8±9.8 years (range, 54 to 90 years). The injuries were sustained from falls in all 16 cases. There were 4 and 5 cases with a history of diabetes and cardiovascular diseases respectively. Surgeries were performed as an emergency after an average duration of 4.4±1.0 h of the injury (Table 1).

**Table 1:** Baseline characteristics

Variable	S-PCP group (n=16)	M-PCP group (n=19)	M-C group (n=14)
Age (year)	64.8±9.8	65.4±9.3	64.6±9.5
Sex			
Male (n)	4 (25%)	5 (26.3%)	4 (28.6%)
Female (n)	12	14	10
Affected side			
Left (n)	11 (68.8%)	12 (63.2%)	10 (71.4%)
Right (n)	5	7	4
Mechanism of trauma			
Falling (n)	16 (100%)	18 (94.7%)	14 (100%)
Accident (n)	0	1	0
AO/OTA fracture classification			
A_2_ (n)	8 (50%)	10 (52.6%)	8 (57.1%)
A_3_ (n)	8	9	6
History of diabetes and cardiovascular disease			
Diabetes (n)	4 (25%)	3 (15.8%)	4 (28.6%)
Cardiovascular disease (n)	5 (31.3%)	6 (31.6%)	6 (42.9%)
Ulnar styloid fracture (n)	8 (50%)	9 (47.4%)	8 (57.1%)
The time from trauma to surgery(treatment) (h)	4.4±1.0	4.5±1.2	3.6±1.1
Surgery duration (min)	55.3±17.2	55.8±17.3	
Follow-up time (month)	30.5±5.1	30.6±5.1	31.9±4.2

Data are presented as a frequency count or mean ± SD. p<0.05 was considered statistically significant. A comparison of data between groups was performed using a one-way analysis of variance (ANOVA), and Least Significant Difference(LSD) was used when making multiple comparisons. Surgery duration between the two groups was performed using a t-test. The chi-square test was used for the comparison of the rest of the characteristics between the three groups

M-PCP group involved 19 patients, 14 female and 5 male cases respectively. Twelve were on the left and 7 on the right, 10 A_2_ types and 9 A_3_ types. Nine were complicated ulnar styloid fractures. The average age was 65.4±9.3 years (range, 52 to 89 years). The injuries were sustained from falls in 18 cases and a vehicular accident in 1 case. This group also had 3 and 6 cases with a history of diabetes and cardiovascular disease respectively. Surgeries were performed as an emergency after an average duration of 4.5±1.2 h of the injury (Table 1).

M-C group involved 14 patients, 10 female and 4 male cases respectively. Ten were on the left and 4 on the right, 8 A2 types and 6 A3 types. Eight were complicated ulnar styloid fractures. The average age was 64.6±9.5 years (range, 53 to 89 years). The injuries were sustained from falls in all 14 cases. This group had 4 and 6 cases with a history of diabetes and cardiovascular disease respectively. Treatment was performed as an emergency after an average duration of 3.6±1.1 h of the injury (Table 1).

**Fig. 1:**
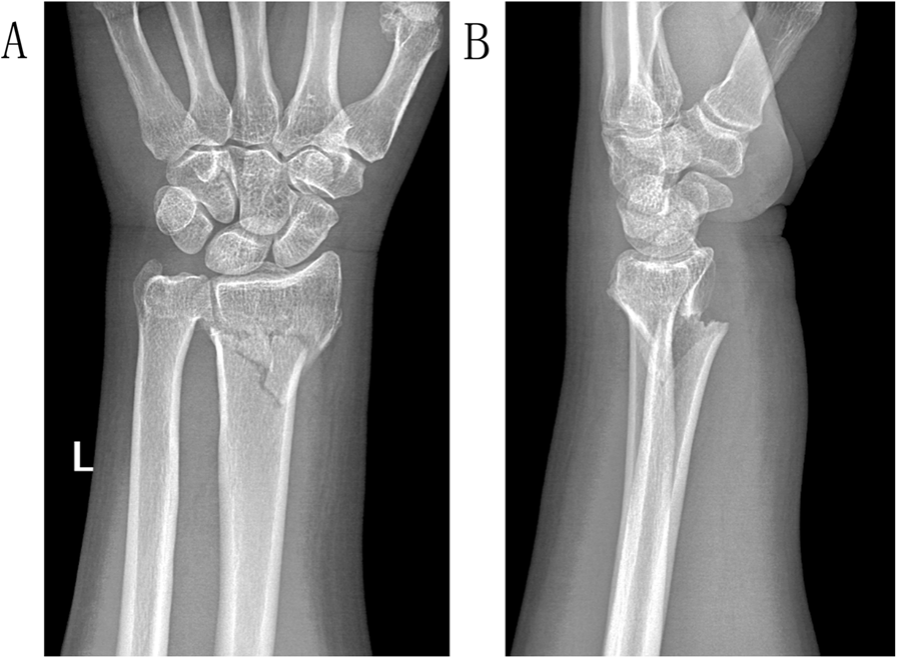
A 65-year-old male patient suffered a trauma to the left wrist when he slipped and fell. The frontal and lateral X-ray films showed distal radius fracture with reduced radial height (11.06mm), radial inclination (19.88°), and volar tilt (−18.80°). The radial shift (+2.37mm) and ulnar variance (+1.74mm) were abnormal as well. AO/OTA classification was A_2_ type

All the patients received frontal and lateral X-ray films (Fig. 1) preoperative (pretreatment). X-ray films were obtained for the unaffected frontal and lateral sides by C-arm fluoroscopy during the surgery (treatment). These X-ray films served as references for the reduction. Patients who had diabetes and cardiovascular disease histories were administered with emergency consultation preoperative.

### Surgical procedure

**Fig. 2:**
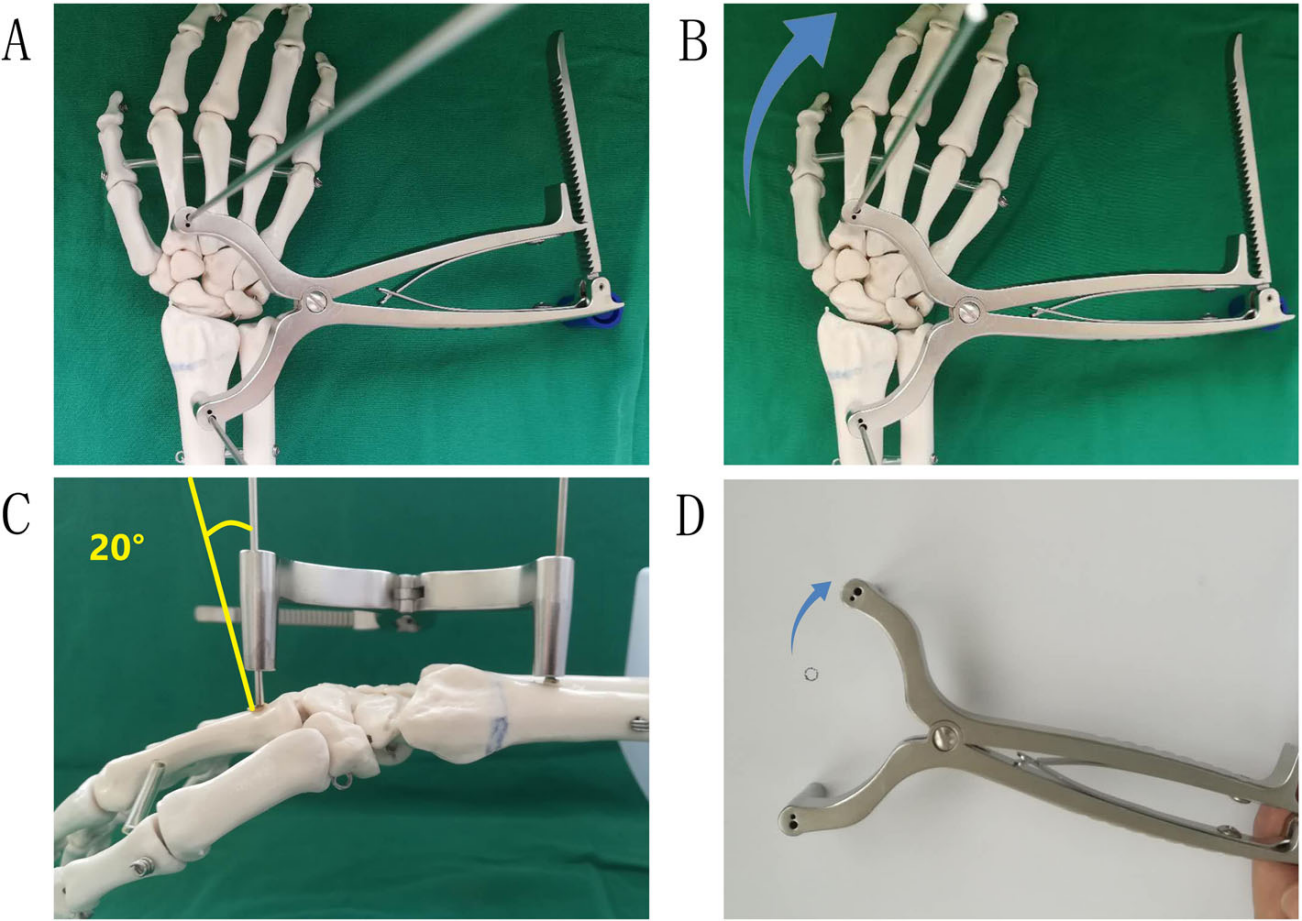
One 2.0 Steinmann pin was drilled into the proximal of the second metacarpal, and the other was drilled into the proximal fragment of fracture (**A**). Gradually distracted the Steinmann pins with the retractor until the height of the radius approximated equal to the unaffected side (**B**). With the retractor opening up like a fan (**B**, **D**), it is not merely providing axial traction but also contributing to increasing the radial inclination. It should be noticed that the Steinmann pin inserted into the proximal of the second metacarpal should maintain a deviation of about 20° from the vertical angle (**C**)

**Fig. 3:**
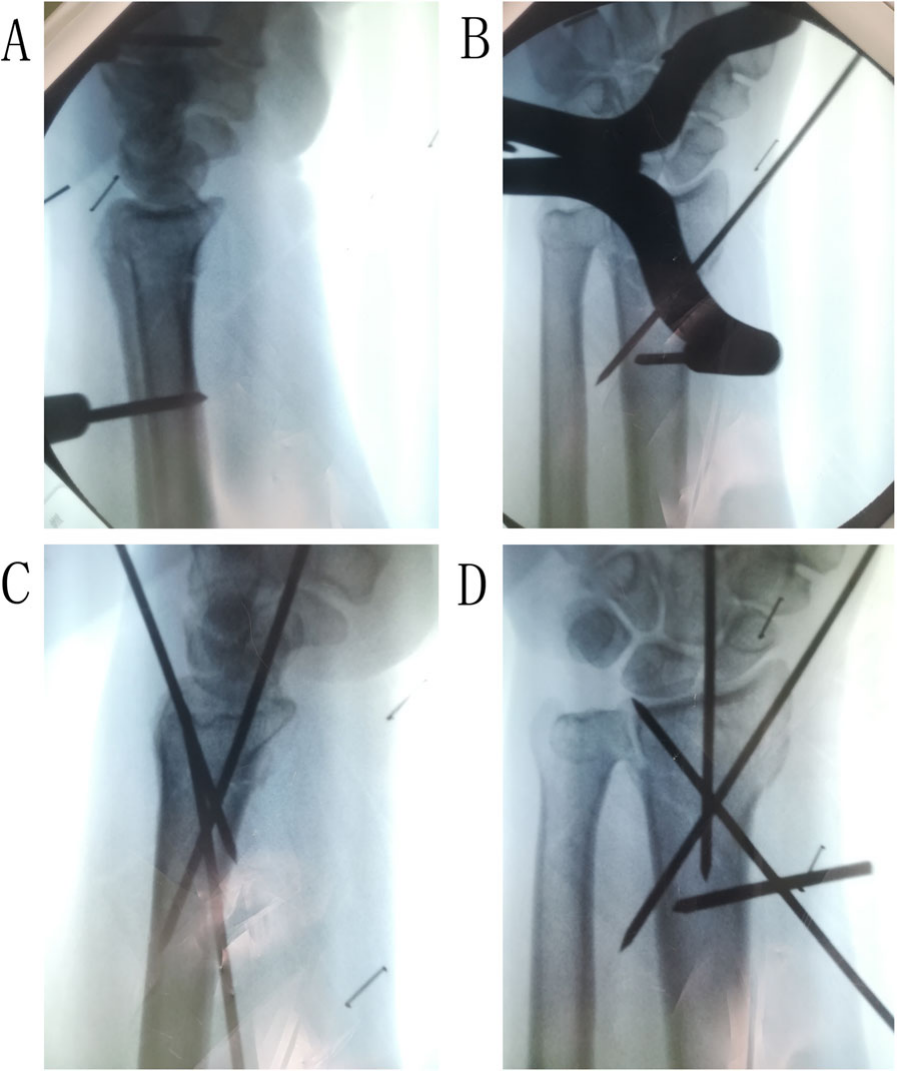
After a sound anatomic reduction was achieved (**A**, **B**), three 1.8 Steinmann pins (Styloid process pin, Lister tubercle pin, Sigmoid notch pin) were inserted in sequence and were expected to achieve a cross-distribution in the frontal and lateral X-ray films (**C**, **D**). The radial height (11.33mm), radial inclination (21.44°), volar tilt (10.32°), the radial shift (+0.26mm), and ulnar variance (−1.22mm) were also confirmed improved (**C**, **D**)

S-PCP was performed under brachial plexus block anesthesia in the supine position. Firstly, the surgical area of the affected upper limb was routinely disinfected and covered with sterile towels on a radiation-permeable surgical table. Secondly, one 2.0 Steinmann pin was drilled into the proximal of the second metacarpal, and the other was drilled into the proximal fragment of the fracture. A Steinmann pin retractor (Huatrau, Chinatrau instrument CO`. Ltd, Guangzhou city, China) (Figs. 2 A and B, and 3 A and B) was used to distract the Steinmann pin until the height of the radius was approximately equal to the unaffected side. This led to the automatic restoration of the radial height, radial inclination, and volar tilt. However, the dorsal displacement or radial shift was restored by squeezing. Thirdly, three 1.8 Steinmann pins were inserted percutaneously from the styloid process of the radius (styloid process pin), the ulnar margin of the Lister tubercle (Lister tubercle pin), and the proximal fragment of the fracture (sigmoid notch pin) in sequence, to achieve a cross-distribution in the frontal and lateral X-ray films (Fig. 3C, D). The retractor and 2.0 Steinmann pins were removed after this process. Fourthly, the wrist was fixed with a cast splint, with the proximal end of the cast not exceeding the elbow and the distal end not exceeding the metacarpophalangeal joint, to facilitate the early exercising of flexion and extension of the fingers.

The M-PCP was performed under the same anesthesia and surgical position. However, the manipulative reduction method was used before the PCP, and the 1.8 Steinmann pins were inserted according to the S-PCP group. Finally, the cast splint was used. The M-C was performed under local anesthesia in the supine position, and the manipulative reduction method was used before the cast splint.

### Postoperative management and evaluation

Non-steroidal anti-inflammatory drugs were administered, and the dressing located at the end of the pins was changed every 3 to 5 days to the patients postoperative. All patients were encouraged to do finger flexion and dorsiflexion actively post-anesthesia. The “R.I.C.E” principle was carried out, aimed at decreasing the swelling. Pins and plaster splints were removed 6 weeks postoperative (posttreatment) until the evidence of the excellent condition of bridging in the fracture end was reflected on the X-ray. Also, full daytime exercises for the wrist were encouraged. They included grip, flexion, and dorsiflexion actively, and the circumduction movements of the wrist with hands passively, aimed at rehabilitating the normal function of the wrist. The affected side was allowed for partial carrying-bearing 10 weeks postoperative with an absence of tapping pain along the axis of the wrist.

### Follow-up

**Fig. 4:**
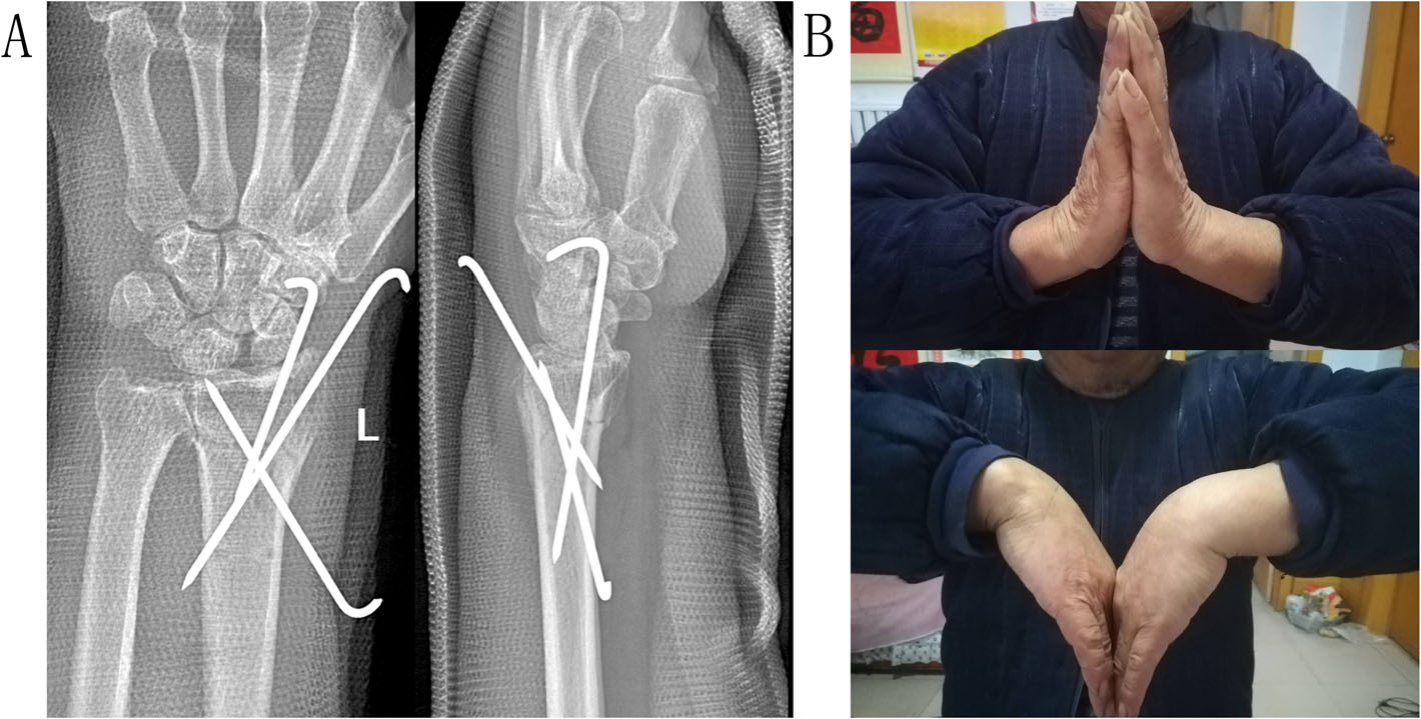
The frontal and lateral X-ray films (**A**) showed evidence of the excellent condition of bridging in the fracture end 6 weeks postoperative. Six months postoperative, the affected wrist achieved a good function (**B**). The VAS score was 0, the modified Mayo wrist score was 80, and the *Quick*DASH score was 15.99

**Fig. 5:**
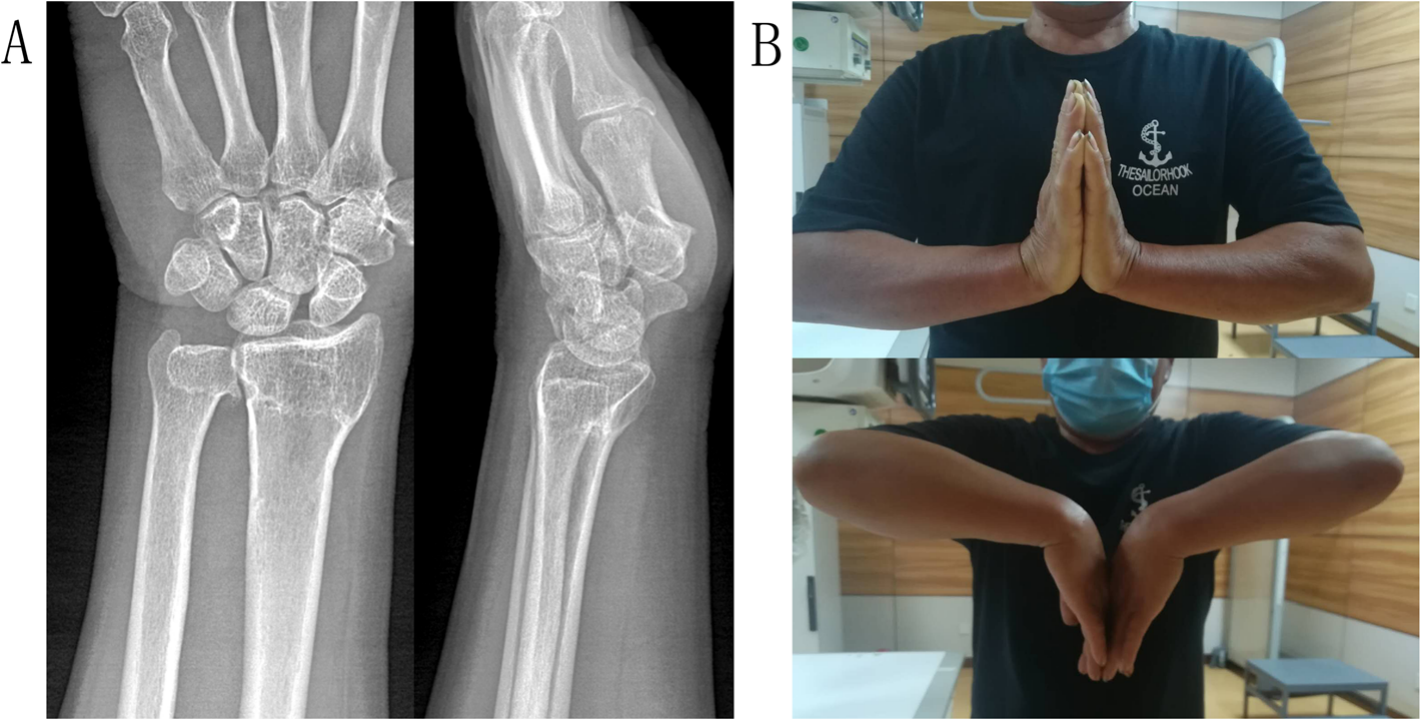
The frontal and lateral X-ray films (**A**) showed evidence of the excellent fracture healing of 26 months postoperative. At the last follow-up, the affected wrist achieved an excellent function (**B**). The VAS score was 0, the modified Mayo wrist score was 95, and the *Quick*DASH score was 2.27

All patients were followed up for a period of 24 to 40 months and were evaluated clinically and radiologically at 8 weeks postoperative (posttreatment), including the last follow-up. The range of wrist motion, including extension, flexion, radial deviation, ulnar deviation, pronation, supination, and grip strength, was measured. The clinical evaluation included a VAS (0=no pain, 10=maximum imaginable pain) score, the modified Mayo wrist score, and the *Quick*DASH score. The radiological parameters, including radial height, radial inclination, ulnar variance, and radial shift, were measured in the frontal X-ray film. The volar tilt was measured in the lateral X-ray film (Figs. 4A and 5A). Radiological parameters were measured by the PACS system (Picture Archiving and Communication Systems, version 2.5, Founder Group, Beijing, China). Clinical evaluation and radiological measurements were carried out by an independent physician who was not involved in the surgeries (treatments).

### Statistical analysis

Where applicable, all data were presented as means ± standard deviation. A comparison of data was performed using a paired t-test for the paired data. A comparison of data between groups was performed using a one-way analysis of variance (ANOVA), and Least Significant Difference (LSD) was used for multiple comparisons. The chi-square test was used for the comparison of the excellent rate. All statistical analyses were performed using the Statistic Package for Social Science (SPSS 22.0). Probability values < 0.05 were considered to be statistically significant.

## Results

The demographic differences between the three groups before surgery (treatment), including age, sex, affected side, mechanism of trauma, AO/OTA fracture classification, the complication of ulnar styloid fracture, diabetes history, cardiovascular disease history, the time from trauma to surgery (treatment), and follow-up time, manifested no statistical significance (Table 1). There was no statistically significant difference between the two groups in surgery duration. All the patients had a normal alignment and stability of the wrist joint without signs of chronic swelling. There was no case of scarring, tendon injury or rupture, vascular injury, nerve injury, pin site infection, or complex regional pain syndrome (CRPS). A patient in the S-PCP group had one pin migration at 4 weeks postoperative: that pin was removed, while the other remaining two pins were left intact which effectively prevented the loss of fracture reduction in the subsequent 2 weeks. At the last follow-up, one patient in the M-PCP group experienced moderate pain after work but did not need long-term use of painkillers; one patient in the M-PCP group also experienced wrist joint stiffness, but no traumatic arthritis emerged at the last follow-up. Two patients in the M-C group experienced moderate pain after work, and one patient with nontraumatic arthritis joint stiffness.

All radiological parameters were significantly improved after surgery (treatment). They were maintained well in the S-PCP and M-PCP groups in the next two follow-ups (Table 2). The patients in the M-C group experienced significant re-displacement at the first and last follow-ups, specifically the radial height and ulnar variance. There were no statistically significant differences between the three groups in the range of wrist motion, grip strength, VAS score, modified Mayo wrist score, and *Quick*DASH score at the first follow-up. The range of wrist motion and clinical evaluation at the last follow-ups (Fig. 5B), including extension, flexion, radial deviation, ulnar deviation, pronation, supination, and grip strength and VAS score, modified Mayo wrist score, and *Quick*DASH score, improved significantly in each group compared to the parameters at the first follow-up (Table 3). More importantly, the radial height (postoperative, 13.33±1.74mm; first follow-up, 13.27±1.81mm; last follow-up, 13.16±1.76mm) and ulnar variance (postoperative, −0.10±1.29mm; first follow-up, −0.05±1.27mm; last follow-up, −0.12±1.09mm) in the S-PCP group improved significantly as compared to the M-PCP and M-C groups. These parameters also manifested no significant difference when compared with the unaffected side. In addition, the range of wrist motion including extension (89.94±5.21%), radial deviation (90.69±6.01%), and supination (90.25±5.87%) improved significantly in the S-PCP group as compared to the M-PCP group; the ulnar deviation (89.81±5.82%) and QuickDASH score (2.70±3.64) in the S-PCP group improved significantly compared to the M-C group; grip strength (92.50±5.59%), pronation (90.50±6.04%), and modified Mayo wrist score (90.94±4.17, the excellent rate reached up to 75%) in the S-PCP group improved significantly as well compared to the M-PCP and M-C groups at the last follow-up (Table 4).

**Table 2:** Radiological parameters

Measure	Group(n)	Unaffected side	Preoperative(pretreatment)	Postoperative(posttreatment)	First follow-up(8 weeks)	Last follow-up
		Mean±sd (95% CI)	Mean±sd (95% CI)	Mean±sd (95% CI)	Mean±sd (95% CI)	Mean±sd (95% CI)
Radial height (mm)	S-PCP (16)	13.96±1.13* (13.36 to 14.56)	9.70±2.80^#^ (8.20 to 11.19)	13.33±1.74* (12.40 to 14.26)	13.32±1.75* (12.39 to 14.25)	13.16±1.76* (12.23 to 14.10)
	M-PCP (19)	13.74±1.19* (12.94 to 14.24)	9.53±2.40^#^ (8.37 to 10.69)	11.78±1.43*^#△^ (11.09 to 12.47)	11.70±1.39*^#△^ (11.02 to 12.37)	11.52±1.41*^#△^ (10.84 to 12.20)
	M (14)	13.35±1.66* (12.40 to 14.31)	9.17±2.12^#^ (7.96 to 10.39)	11.46±1.35*^#△^ (10.68 to 12.24)	10.65±1.17*^#△^ (9.98 to 11.33)	10.31±1.07^#△^ (9.68 to 10.93)
Radial inclination (°)	S-PCP (16)	26.56±2.95* (25.00 to 28.14)	19.24±5.43^#^ (16.35 to 22.13)	26.16±3.89* (24.08 to 28.23)	25.84±4.07* (23.67 to 28.00)	25.27±4.22* (23.02 to 27.52)
	M-PCP (19)	26.04±1.71* (25.53 to 26.96)	19.17±5.23^#^ (16.30 to 22.33)	24.27±3.36*^#^ (22.97 to 26.44)	24.14±3.41*^#^ (22.90 to 26.30)	23.73±3.34*^#^ (22.12 to 25.34)
	M (14)	26.05±3.58* (23.97 to 28.12)	19.31±5.30^#^ (16.24 to 22.37)	24.07±3.34*^#^ (22.15 to 26.00)	23.60±3.28*^#^ (21.70 to 25.50)	23.20±3.36*^#^ (21.26 to 25.15)
Radial shift (mm)	S-PCP (16)	0	2.57±2.86 (1.05 to 4.10)	0.46±0.56* (0.15 to 0.65)	0.41±0.48* (0.15 to 0.66)	0.39±0.47* (0.14 to 0.64)
	M-PCP (19)	0	1.98±2.17 (0.93 to 3.03)	0.84±1.32* (0.21 to 1.47)	0.82±1.29* (0.20 to 1.44)	0.80±1.29* (0.18 to 1.42)
	M (14)	0	2.73±2.16 (1.47 to 3.98)	0.75±0.77* (0.30 to 1.19)	0.75±0.76* (0.31 to 1.19)	0.74±0.75* (0.30 to 1.16)
Volar tilt (°)	S-PCP (16)	11.67±2.63* (10.28 to 13.07)	−22.15±11.37^#^ (−28.21 to −16.09)	7.36±6.91*^#^ (3.68 to 11.04)	7.23±6.94*^#^ (3.53 to 10.92)	6.49±7.01*^#^ (2.76 to 10.23)
	M-PCP (19)	11.75±2.40* (11.00 to 12.90)	−22.46±14.10^#^ (−29.25 to −15.66)	6.49±5.70*^#^ (3.74 to 9.24)	6.35±5.79*^#^ (3.56 to 9.14)	5.68±5.82*^#^ (2.87 to 8.48)
	M (14)	11.04±3.16* (9.22 to 12.87)	−21.70±10.41^#^ (−27.71 to −15.69)	7.03±2.43*^#^ (5.63 to 8.44)	2.08±2.48*^#△^ (0.65 to 3.51)	1.35±2.47*^#△^ (−0.08 to 2.77)
Ulnar variance (mm)	S-PCP (16)	−0.26±0.69* (−0.61 to 0.10)	2.17±2.40^#^ (0.89 to 3.45)	−0.08±1.23* (−0.57 to 0.74)	−0.05±1.27* (−0.72 to 0.63)	−0.12±1.09* (−0.70 to 0.46)
	M-PCP (19)	−0.30±0.63* (−0.61 to 0.00)	3.41±3.45^#^ (1.75 to 5.08)	1.25±1.93*^#△^ (−0.32 to 2.18)	1.19±1.94*^#△^ (0.25 to 2.12)	1.19±1.99*^#△^ (0.23 to 2.14)
	M (14)	−0.31±0.59* (−0.65 to −0.03)	2.87±2.65^#^ (1.34 to 4.40)	1.31±1.75*^#^ (−0.30 to 2.32)	1.99±1.89^#△^ (0.90 to 3.08)	2.01±1.89^#△^ (0.92 to 3.10)

Data are presented as mean±SD. p<0.05 is considered statistically significant. A comparison of data was performed using a paired t-test for paired data (*p<0.05 vs. preoperative, ^#^p<0.05 vs. unaffected side). A comparison of data between groups was performed using a one-way analysis of variance (ANOVA), and Least Significant Difference(LSD) was used when making multiple comparisons (^△^p<0.05 vs. S-PCP group). The Welch method was used when the variances were unequal, and the Tamhane method was used for multiple comparisons

**Table 3:** The range of wrist motion, grip strength, and clinical results

Measure	Group (n)	First follow-up (8 weeks)	Last follow-up
		Mean±sd (95% CI)	Mean±sd (95% CI)
Extension (%)	S-PCP (16)	35.44±7.76 (31.30 to 39.57)	89.94±5.21* (87.16 to 92.71)
	M-PCP (19)	33.05±9.19 (28.93 to 38.57)	82.58±11.19*^%^ (77.18 to 87.97)
	M (14)	36.64±8.58 (31.69 to 41.60)	82.29±13.02* (74.77 to 89.80)
Flexion (%)	S-PCP (16)	35.44±6.97 (31.73 to 39.14)	89.38±7.37* (85.44 to 92.30)
	M-PCP (19)	33.11±7.95 (29.27 to 36.94)	83.89±10.69* (78.74 to 89.05)
	M (14)	36.36±8.44 (31.49 to 41.23)	82.50±13.29* (74.82 to 90.18)
Radial deviation (%)	S-PCP (16)	33.13±7.17 (29.30 to 36.95)	90.69±6.01* (87.56 to 93.06)
	M-PCP (19)	29.11±10.31 (24.13 to 34.07)	83.26±9.07*^%^ (78.56 to 87.50)
	M (14)	33.64±6.49 (29.90 to 37.39)	83.57±13.74* (75.64 to 91.50)
Ulnar deviation (%)	S-PCP (16)	34.25±7.13 (30.45 to 38.05)	89.81±5.82* (86.71 to 92.91)
	M-PCP (19)	30.74±7.46 (27.14 to 34.33)	83.95±8.52* (79.84 to 88.05)
	M (14)	32.50±7.12 (28.39 to 36.61)	82.79±12.72*^%^ (75.44 to 90.13)
Grip strength (%)	S-PCP (16)	37.88±6.81 (34.25 to 41.50)	92.50±5.59* (89.52 to 95.48)
	M-PCP (19)	33.11±9.77 (28.79 to 38.96)	85.89±8.53*^△^ (81.78 to 90.00)
	M (14)	34.64±5.87 (31.25 to 38.04)	86.29±10.21*^△^ (80.39 to 92.18)
Pronation (%)	S-PCP (16)	32.63±6.48 (29.17 to 36.08)	90.50±6.04* (87.28 to 93.72)
	M-PCP (19)	32.32±8.49 (27.90 to 37.48)	84.74±7.99*^△^ (80.88 to 88.59)
	M (14)	31.86±5.39 (28.74 to 34.97)	84.35±8.47*^△^ (79.46 to 89.25)
Supination (%)	S-PCP (16)	32.25±8.10 (27.94 to 36.56)	90.25±5.87* (87.12 to 93.37)
	M-PCP (19)	29.89±9.12 (25.49 to 34.29)	82.11±8.54*^%^ (77.99 to 86.22)
	M (14)	32.64±7.62 (28.24 to 37.04)	83.71±11.07* (77.32 to 90.11)
VAS score	S-PCP (16)	3.31±0.95 (2.81 to 3.82)	0.11±0.60* (0.00 to 0.63)
	M-PCP (19)	3.16±1.07 (2.64 to 3.67)	0.68±0.75* (0.32 to 1.05)
	M (14)	2.93±0.92 (2.40 to 3.46)	0.57±0.85* (0.08 to 1.06)
Modified Mayo wrist score	S-PCP (16)	25.94±4.91 (23.45 to 28.55)	90.94±4.17* (88.71 to 93.16)
	M-PCP (19)	19.74±8.89^%^ (15.45 to 24.02)	79.21±15.39*^%^ (71.79 to 86.63)
	M (14)	28.21±6.96 (24.19 to 32.23)	78.93±12.12*^%^ (71.93 to 85.92)
*Quick*DASH score	S-PCP (16)	50.42±6.22 (47.11 to 53.74)	2.70±3.64* (0.76 to 4.64)
	M-PCP (19)	54.55±9.09 (50.16 to 58.93)	7.54±8.02* (3.67 to 11.40)
	M (14)	49.35±7.69 (44.91 to 53.79)	8.93±7.80*^%^ (4.43 to 13.43)

Data are presented as mean±SD. p<0.05 is considered statistically significant. A comparison of data was performed using paired t-test for paired data (*p<0.05 vs. first follow-up). A comparison of data between groups was performed using a one-way analysis of variance (ANOVA), and Least Significant Difference (LSD) was used when making multiple comparisons (^△^p<0.05 vs. S-PCP group). The Welch method was used when the variances were unequal, and the Tamhane method was used for multiple comparisons (^%^p<0.05 vs. S-PCP group)

**Table 4:** The excellent ratio of the modified Mayo wrist score

Group (n)	First follow-up (8 weeks)				Last follow-up			
	Excellent	Good	Fair	Poor	Excellent	Good	Fair	Poor
S-PCP (16)	0	0	0	16	12*	4	0	0
M-PCP (19)	0	0	0	19	5*^△^	9	3	2
M (14)	0	0	0	14	4*^△^	5	2	3

Data are presented as row x column table information. p<0.05 is considered statistically significant. The chi-square test was used for the comparison of the excellent rate between the three groups and the difference between the two follow-up periods in the same group (^△^p<0.05 vs. S-PCP group, *p<0.05 vs. first follow-up)

## Discussion

The primary goals for the treatment of elderly distal radius fractures are painlessness and perfect wrist function. To achieve these goals, two prerequisites are needed: a sound anatomical reduction and the protection of the fragile soft tissue surrounding the fracture end. Conservative treatment, that is, manipulative reduction combined with immobilization, provides excellent results in an undisplaced fracture. However, for unstable fractures, conservative treatment poses the risk of re-displacement posttreatment [4], and thereby, affected the wrist function. At present, the surgical protocol is still the mainstream for unstable fractures. The external fixator, which avoids open reduction, seems to satisfy both prerequisites. However, CRPS and finger stiffness development can occur if there is a prolonged application of excessive traction [5]. With extensive use of the volar plate [6, 7], ORIF has become the mainstream method but has come with huge medical costs and soaring hospitalization rates [8]. Not to mention, the potential risk of incision complications associated with the fragile soft tissue in the elderly, which potentially retards the rehabilitation of wrist function. Suggestively, isn’t the door still widely opened for practitioners to opt for PCP for the elderly?

M-PCP for the treatment of distal radial fractures has a long history [9, 10]. The mechanism is based on the principle of ligamentotaxis [11]. PCP can only be done after a satisfactory reduction. A sound reduction including the height of the radius and the volar tilt contributes to neutralizing the deforming forces. In particular, brachioradialis tendons, the only tendon attached to the distal fragment, plays an essential role in maintaining a sound reduction and prevention of re-displacement [12]. In the case of poor reduction, the tension of the brachioradialis tendon forces fracture re-displacement and migrates the pin postoperatively. Besides, loss of radial height over 5mm distorts the triangular fibrocartilage complex (TFCC) and affects the stability of the distal radioulnar joint (DRUJ) [13, 14]. This even causes the loss of inclination or volar tilt [15]. Ulnar variance, which reflects the matching relationship between the semilunar bone and sigmoid notch, affects the primary load-bearing intermediate column [16] of the wrist. Either negative or positive values (<−2mm or >2mm) are associated with a wrist degenerate disease or TFCC damage [17]. At present, M-PCP still resorts to pure manipulative reduction technique [10], though a few kinds of literature mentioned pinning pry pulling technology [18, 19]. In this situation, the manipulative reduction method could not satisfy such a high reduction requirement, not to mention maintaining a sound reduction before PCP. It was not until we applied the Steinmann pin retractor that this problem was resolved. Several literature reviews [20, 21] involving the M-PCP have had reflective clinical and radiological results similar to our M-PCP group. However, the S-PCP group had more advantages in our study. To be more precise, these results were approximately equal to the unaffected side.

The Steinmann pin retractor was initially used in the surgical procedure of calcaneal fractures [22]. Several critical techniques need to be observed in using this method for distal radial fractures: Firstly, the 2.0 pin inserted into the proximal fragment should be perpendicular to the shaft, while the other inserted into the second metacarpal should maintain a deviation of about 20° from the vertical angle (Fig. 2C). After installing the retractor, the second 2.0 pin will be passively parallel to the previous one. The wrist will present a palmar flexion passively with the retraction, then the dorsal or palmar deformity, and the volar tilt gets corrected automatically. Secondly, the opening side of the retractor should be towards the radial side (Fig. 2A, B), for the retractor to open up like a fan (Fig. 2B, D). This contributes to increasing the radial inclination. Thirdly, a small incision combined with a pin sleeve can avoid soft tissue injury, likewise, moistening the pins with normal saline prevents the soft tissue entanglement. Fourthly, the tip of the Sigmoid notch pin is recommended to reach the subchondral bone, but not allowed to penetrate the articular surface. That pin is valued merely not because it provides support for the intermediate column [16] but also because it prevents the distal fragment from rotating.

Multiple manipulative reductions damage the reduction markers. It makes it more challenging to obtain an anatomic reduction. Besides, it also tends to damage the periosteal periosteum and the soft tissue surrounding the fracture end, which usually causes aggravated hematoma and bone marrow extrusion. This leads to tendon adhesion and overall stiffness [23], which in turn retards the recovery of wrist function. With the assistance of the retractor, multiple manipulative reductions during the surgery could be avoided, hence producing such a good clinical result. In our study, the S-PCP group had a distinct advantage in the improvement of radial height, ulnar variation, the range of wrist motion, the modified Mayo wrist score (including the excellent rate). No CRPS emerged postoperatively and was ascribed to the short duration of traction during surgery which differs from the long-term use of an external fixator.

This new protocol (S-PCP) does not apply to all kinds of distal radial fractures. The indications include A_2_ and A_3_ types of distal radius fractures in the elderly with limited dorsal comminution, including intra-articular fractures with displacement less than 2mm. The contraindications include intra-articular fractures with a displacement of more than 2mm, oblique volar fractures, die-punch fractures, or dorsal comminution involving more than one-third of the diameter of the articular surface; besides, are patients with severely comminuted fracture, especially at the pinning inserted area, since the risk of pinning migration leads to fracture re-displacement [24].

## Conclusion

S-PCP does not merely contribute to improving fracture reduction but also provides a good wrist function and clinical efficacy in the nutshell. Our present study suggests that this new protocol may serve as an effective method for A_2_ and A_3_ type of distal radius fractures in the elderly with limited dorsal comminution, including intra-articular fractures with displacement less than 2mm.

## Supplementary Information

**Additional file 1.**

## Data Availability

The datasets used and analyzed during the current study are available from the corresponding author on reasonable request.

## Abbreviations

VAS: Visual analog scale
PCP: Percutaneous pinning fixation
S-PCP: Steinmann pin retractor-assisted reduction combined with PCP
M-PCP: Manipulative reduction combined with PCP
M-C: Manipulative reduction combined with cast splint
QuickDASH: Quick Disabilities of the Arm, Shoulder, and Hand
ORIF: Open reduction and internal fixation
AAOS: American Academy of Orthopaedic Surgeons
CRPS: Complex regional pain syndrome
PACS: Picture Archiving and Communication Systems
SPSS: Statistic Package for Social Science
TFCC: Triangular fibrocartilage complex
DRUJ: Distal radioulnar joint
