# Supplementary material for: A novel retractor-assisted closed reduction combined with percutaneous pinning fixation for the treatment of elderly distal radius fractures: a retrospective cohort study
Source: J Orthop Surg Res. 2021 Jun 26;16:409. doi: 10.1186/s13018-021-02556-6 (PMC8235814; doi:10.1186/s13018-021-02556-6)
Supplement: Supplementary file 1 — Additional file 1. [file 13018_2021_2556_MOESM1_ESM.doc]

**Supplementary information**

1. **Ethics approval and consent to participate**

**
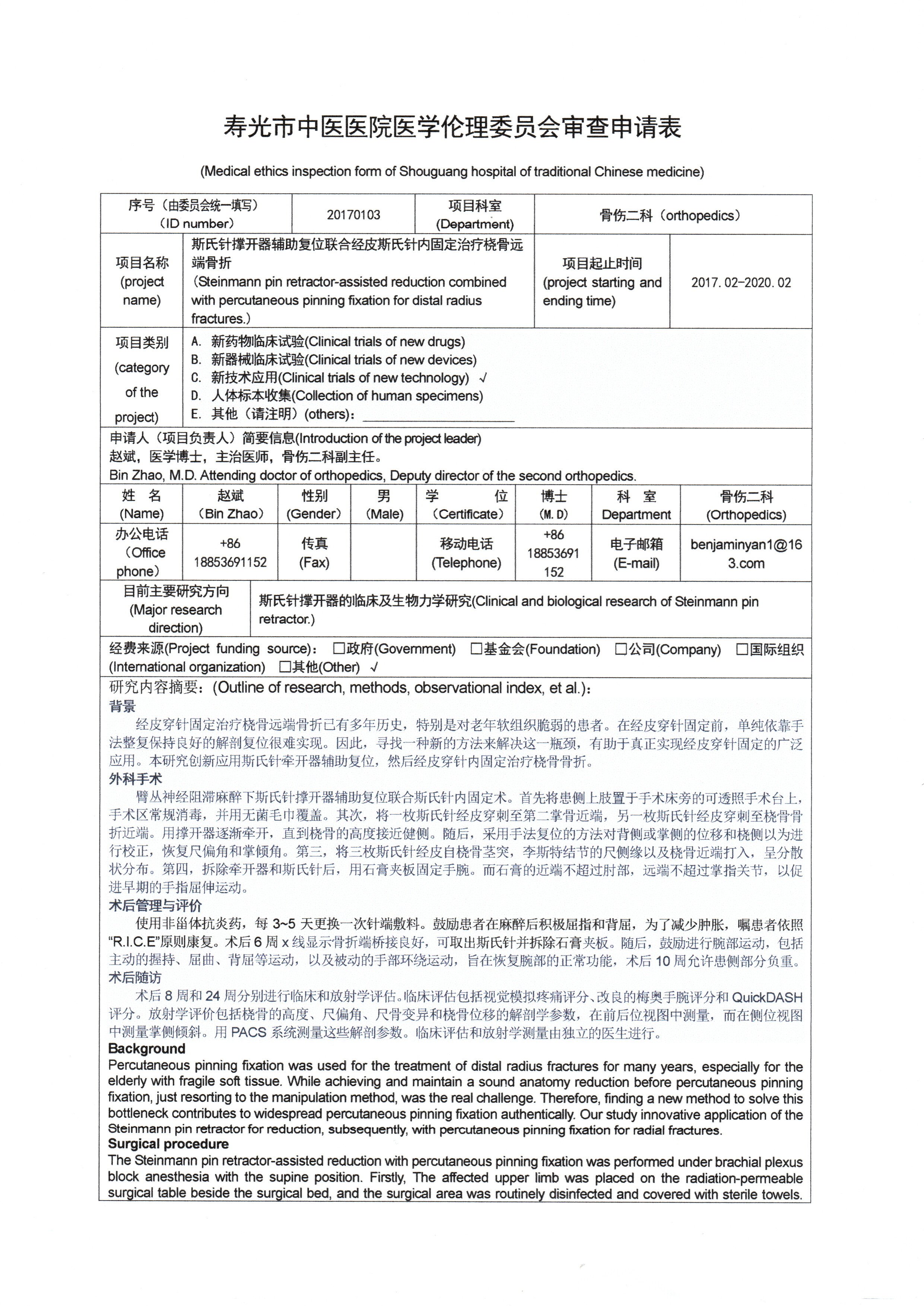
**

**
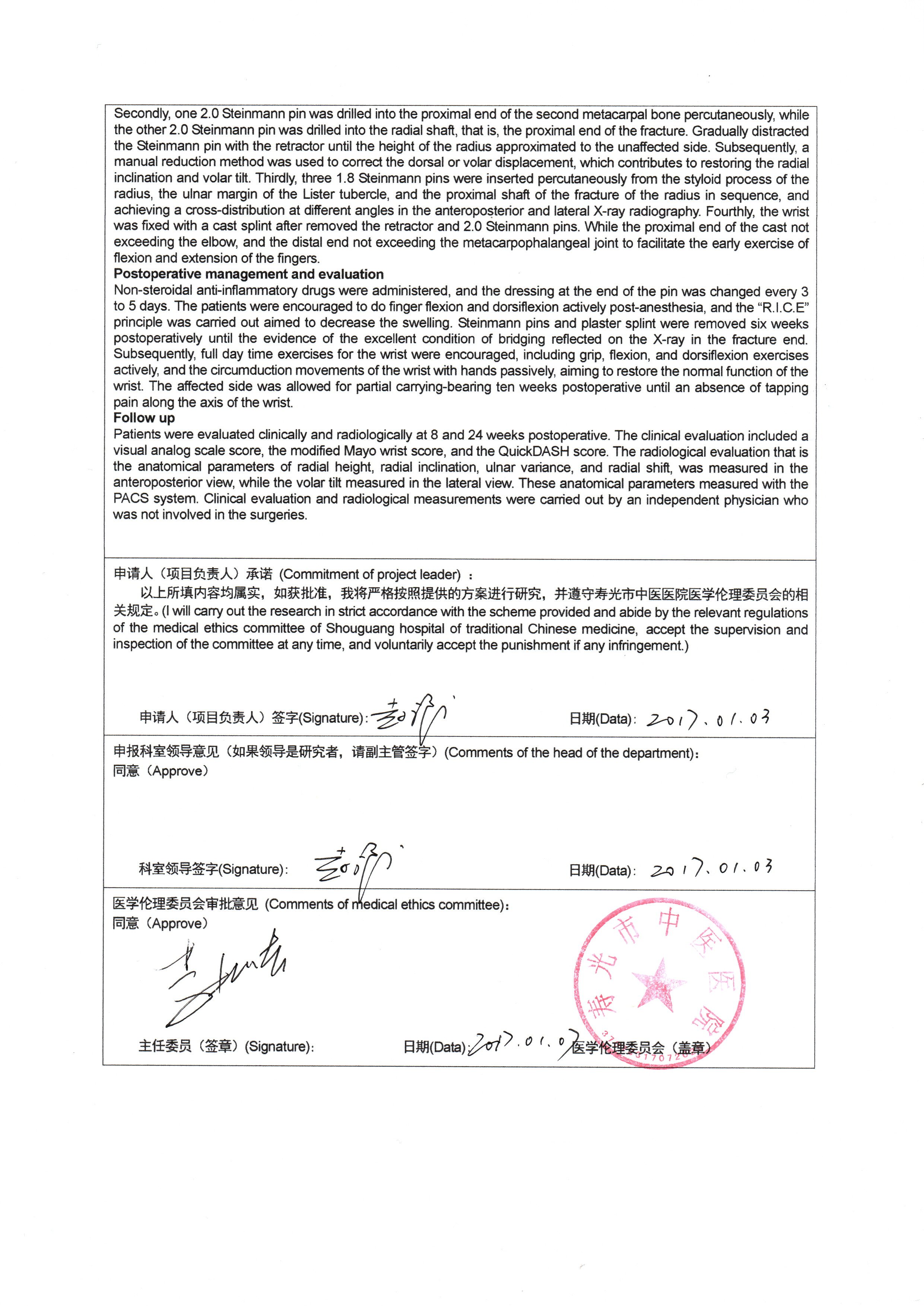
**

**
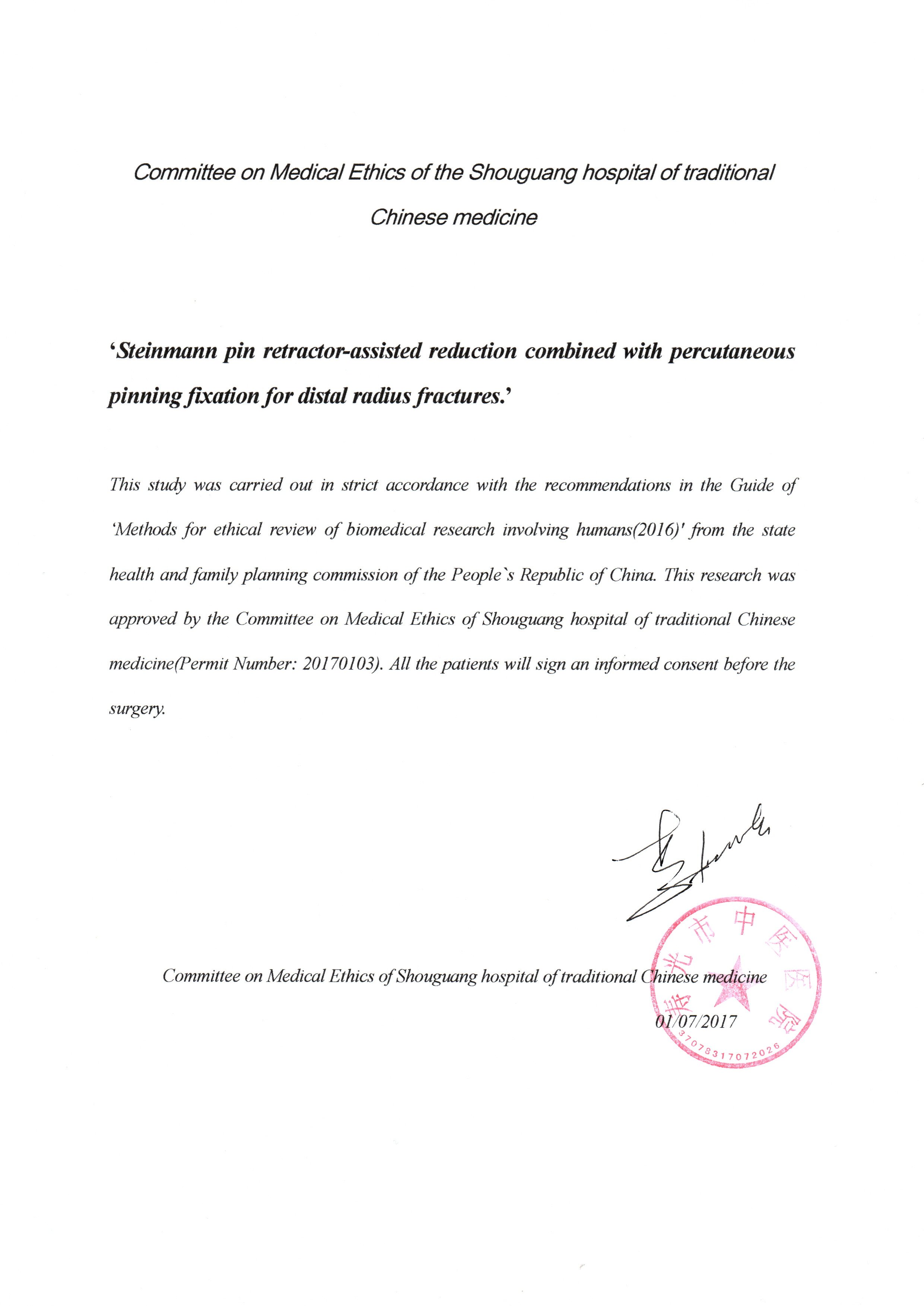
**

1. **informed consent for patient**

**
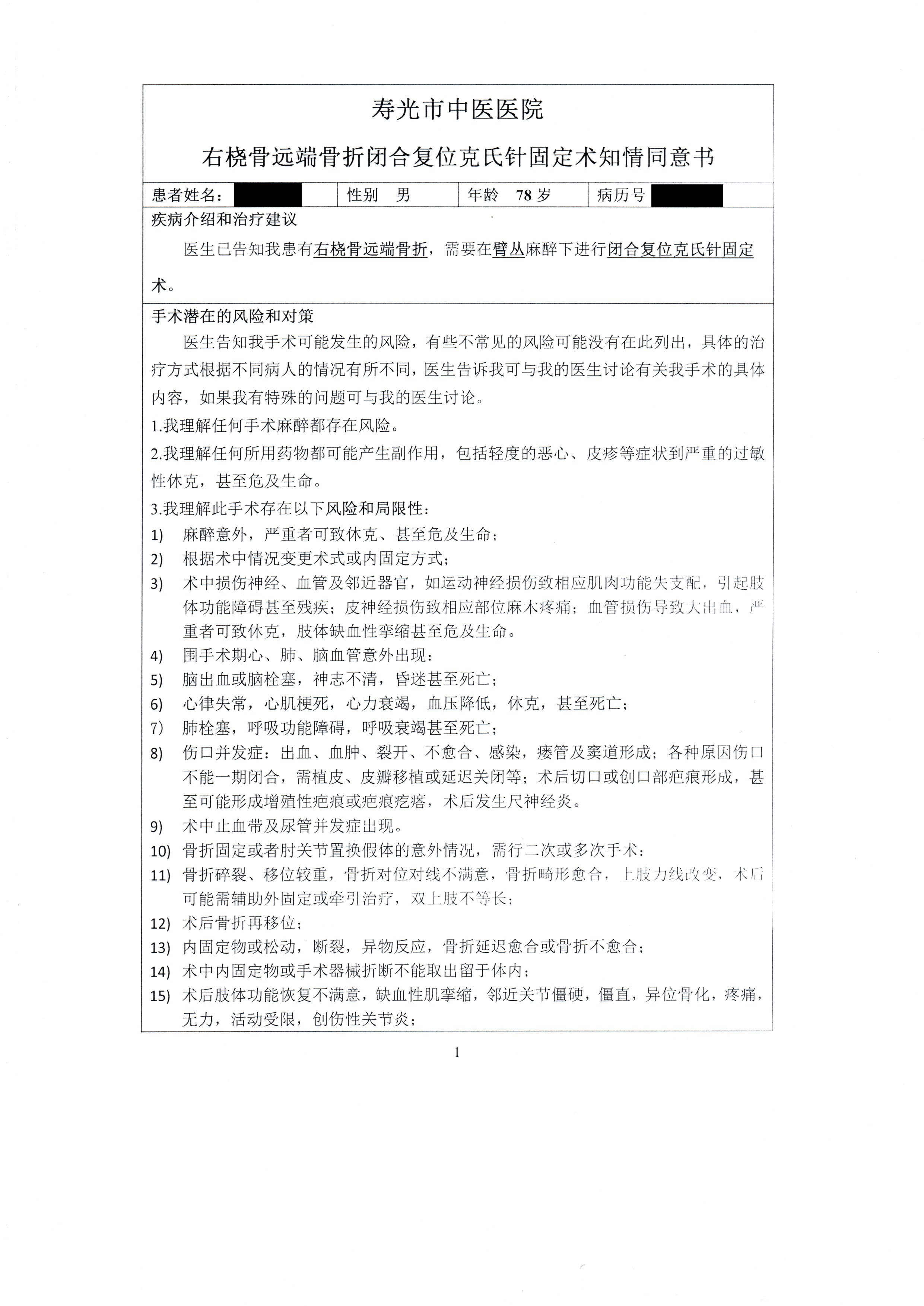
**

**
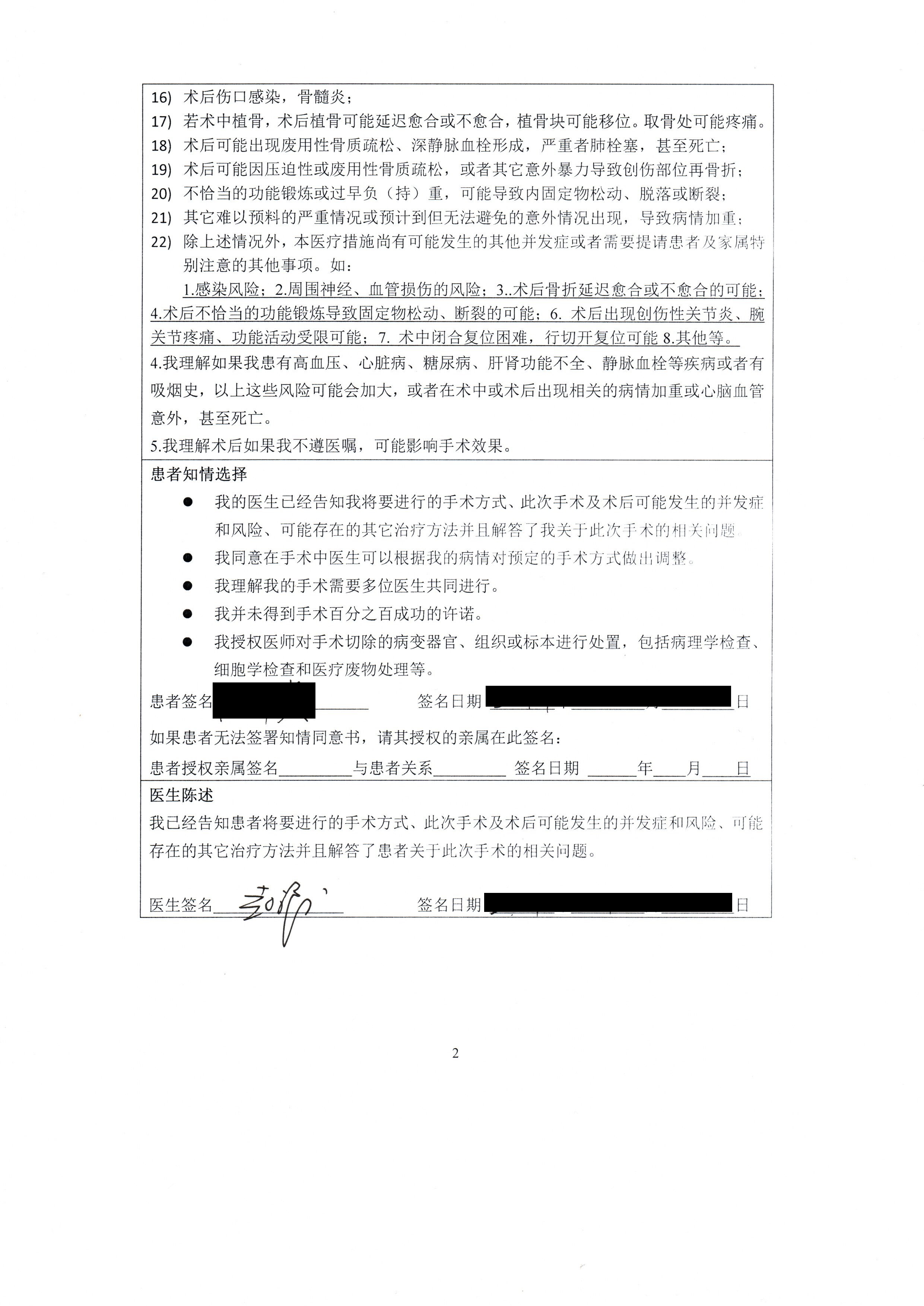
**

**
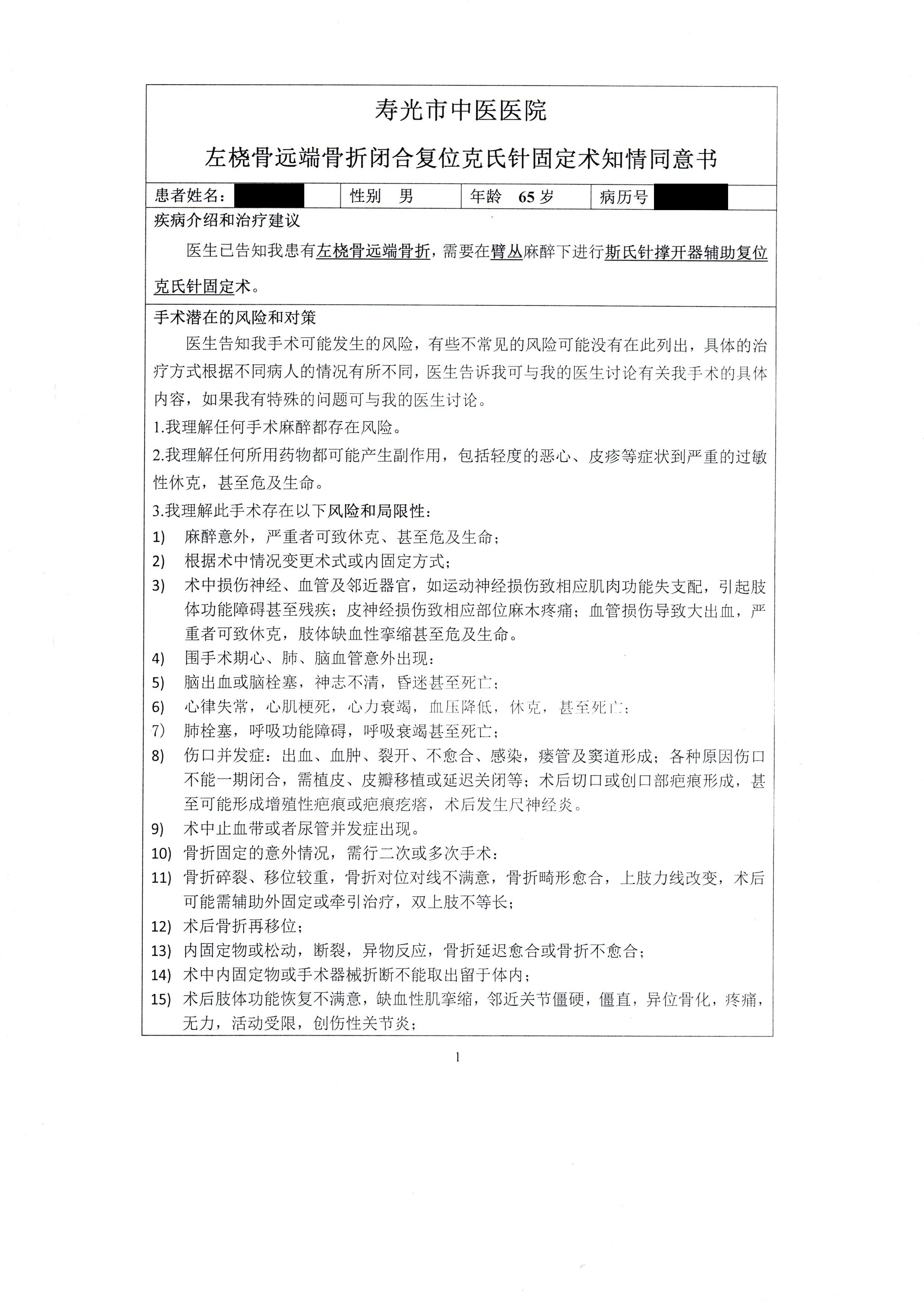
**

**
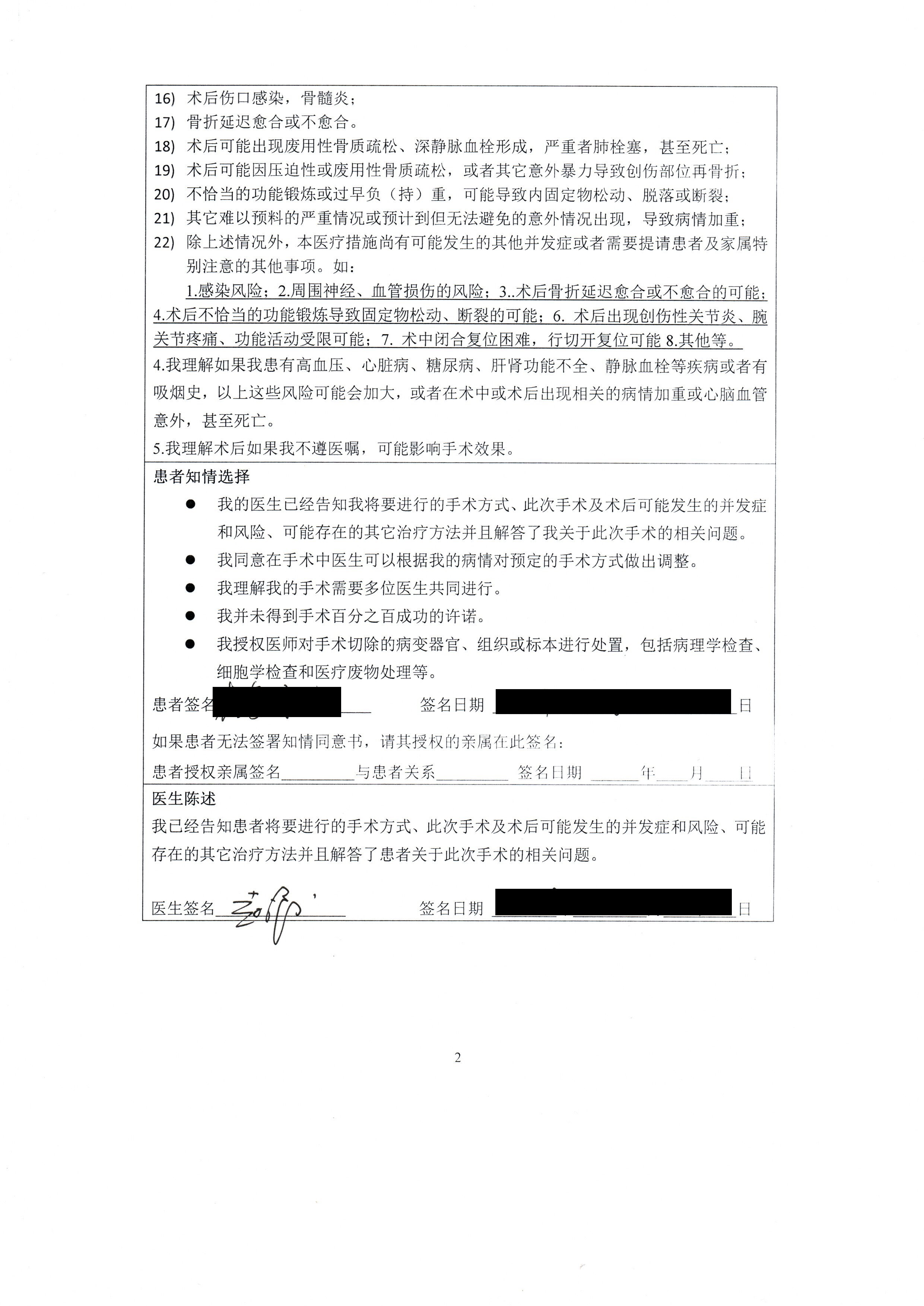
**

1. **Authorization for the use of the right of portrait**

**
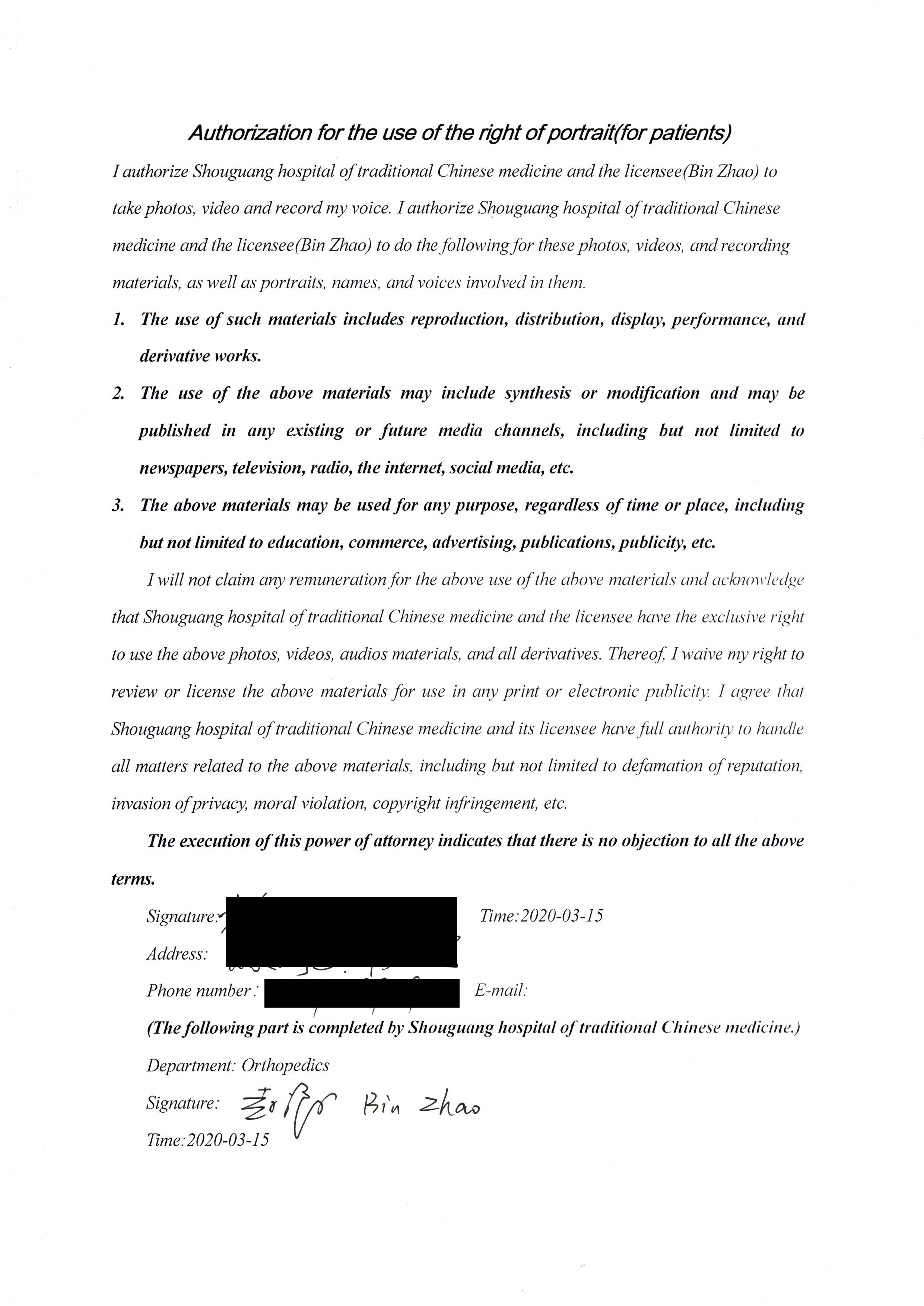
**

**
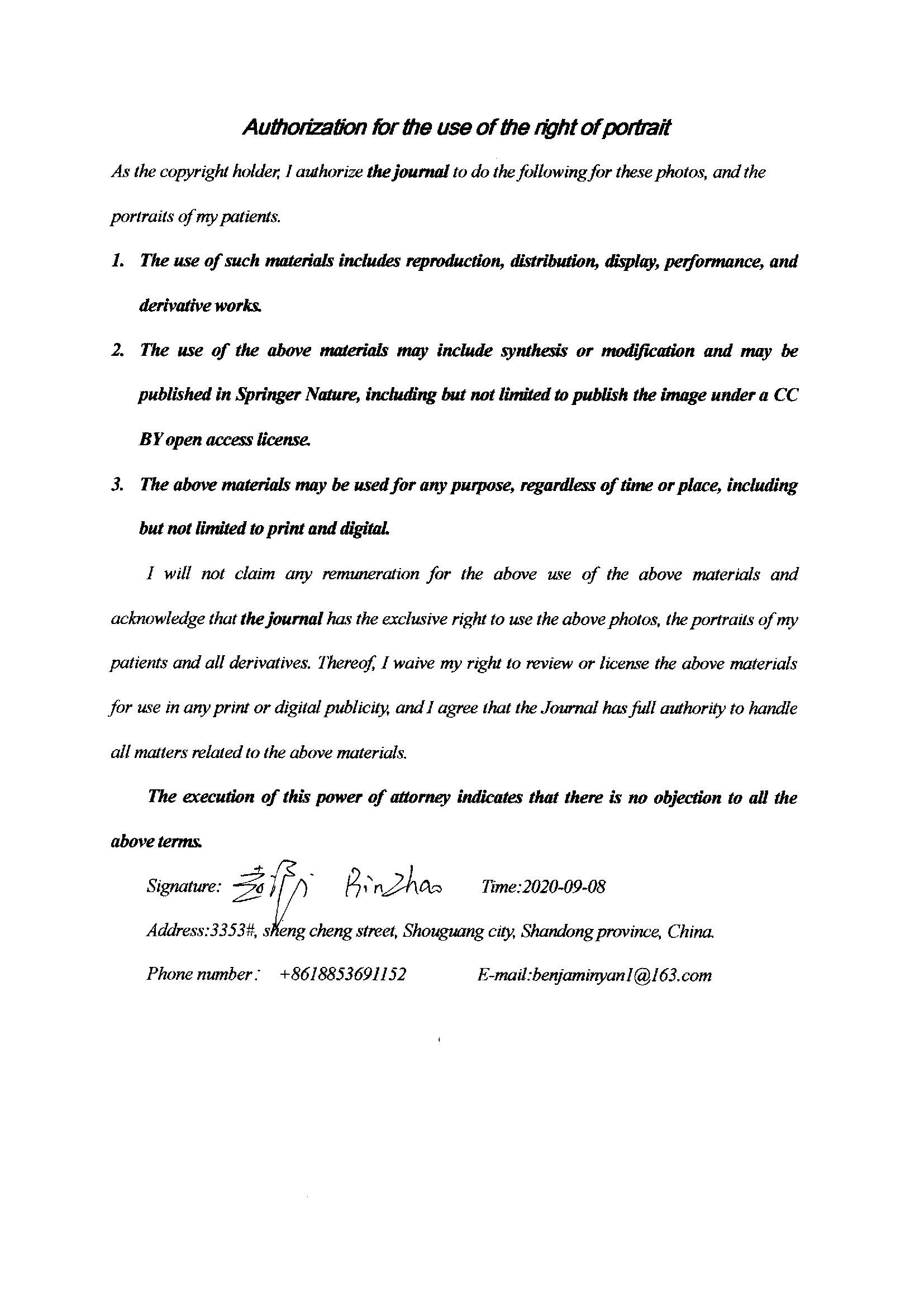
**

**International Journal of Surgery Author Disclosure Form**

The following additional information is required for submission. Please note that failure to respond to these questions/statements will mean your submission will be returned. If you have nothing to declare in any of these categories, then this should be stated.

**Please state any conflicts of interest**

| none |
| --- |

**Please state any sources of funding for your research**

| none |
| --- |

**Please state whether Ethical Approval was given, by whom and the relevant Judgement’s reference number**

| This study was approved by the Committee on Medical Ethnic of Shouguang Hospital of Traditional Chinese Medicine(Permit number: 20170103) and was carried out in strict accordance with the recommendations in the Guide of ‘Methods for ethical review of biomedical research involving humans(2016)’ from the state health and family planning commission of the People’ s Republic of China. The patient provided written, informed consent for the surgery. |
| --- |

**Research Registration Unique Identifying Number (UIN**)

Please enter the name of the registry, the hyperlink to the registration and the unique identifying number of the study. You can register your research at [http://www.researchregistry.com](http://www.researchregistry.com/) to obtain your UIN if you have not already registered your study. This is mandatory for human studies only.

| 1. Name of the registry:   Steinmann pin retractor-assisted reduction combined with percutaneous pinning fixation for distal radius fractures: A prospective cohort study.   1. Unique Identifying number or registration ID:   researchregistry5509   1. Hyperlink to the registration (must be publicly accessible):   https://www.researchregistry.com/register-now#home/registrationdetails/5e96a4f9f0c3b70018ef94d3/ |
| --- |

**Author contribution**

Please specify the contribution of each author to the paper, e.g. study design, data collections, data analysis, writing. Others, who have contributed in other ways should be listed as contributors.

| Conceptualization: Bin Zhao, Rongxiu Bi. Performed the surgery: Bin Zhao. Data curation: Wenqian Zhao. Formal analysis: Wenqian Zhao, Zhenji Li. Original draft: Bin Zhao. An article reviewing, editing, and diction analysis: Isaac Assan. |
| --- |

**Guarantor**

The Guarantor is the one or more people who accept full responsibility for the work and/or the conduct of the study, had access to the data, and controlled the decision to publish. Please note that providing a guarantor is compulsory.

| Bin Zhao; Wenqian Zhao; Rongxiu Bi; |
| --- |
